# CBCT Evaluation of Bony Nasal Pyramid Dimensions in Iranian Population: A Comparative Study with Ethnic Groups

**DOI:** 10.1155/2014/819378

**Published:** 2014-09-18

**Authors:** Asieh Zamani Naser, Mariyya Panahi Boroujeni

**Affiliations:** Department of Oral and Maxillofacial Radiology, School of Dentistry, Isfahan University of Medical Sciences, Esfahan, Iran

## Abstract

*Background*. The aim of the present study was to have normative data of nasal bone thickness for use before reconstructive surgery and nasal augmentation through radiography analysis. *Methods and Materials*. In this descriptive-analytical study, 74 patients were selected from people referred to Radiology Department of Isfahan University for CBCT examination in 2012. Patients with a history of nasal surgery or facial trauma and known congenital anomaly were excluded from the study. Height of nasal bone and width of pyriform aperture and nasal bone thickness in lateral and medial osteotomy line were measured. All these measurements were repeated by two radiologists; finally one sample *t*-test was performed. *Results*. The mean thickness of nasal bone on the lateral osteotomy line was 1.92 ± 0.29 mm in females and 1.73 ± 0.32 mm in males (*P* value = 0.39). The mean thickness of medial osteotomy line was 1.63 ± 0.47 mm in females and 1.94 ± 0.19 mm in males (*P* value = 0.31). The mean length of nasal bone was 23.5 ± 3.34 mm in females and 25.7 ± 2.96 mm in males (*P* value = 0.11). The mean width of pyriform aperture was 23.77 ± 2.58 mm in females and 25.67 ± 1.79 mm in males (*P* value = 0.25). *Conclusions*. The dimensions of nasal pyramid are known to be significant in choosing suitable osteotome size for reducing surgery side effect. Our results can be used for preoperative estimation of nasal bone dimension of people undergoing reconstructive surgery and augmentation.

## 1. Introduction

Nasal bone is an important structure in the shape of entire nose. It is bordered superiorly by the frontal bone, laterally by the maxillary bones, and inferiorly by the pyriform aperture [1]. The size and morphology of nasal bone vary between different races, ethnic groups, genders, and ages [2]. Nasal bone and pyriform aperture can be examined by a physical and X-ray examination and also the three-dimensional computed tomography [1, 3]. Objective data cannot be obtained in this regard, since the result of physical examination can be different between clinicians [1]. Three-dimensional (3D) CT is a perfect technique for obtaining objective data and can be used as a noninvasive preoperative examination [1, 3]. This technique provides advanced information about the craniofacial anatomic anomalies, paranasal sinuses, and nasal cavity, as well as dental structures [3]. Nowadays, the use of three-dimensional radiographies (like cone beam computed tomography) is increasing for diagnosis and treatment in different fields of dentistry [4, 5], such as implant dentistry, maxillofacial surgery [6, 7], orthodontics [8], and endodontics [9], in comparing the craniofacial growth of patients with anomalies, and in planning reconstructive surgery on the craniofacial region [1]. The normative data for the bony nasal pyramid dimension among ethnic and gender groups could provide credible references for the estimation of optimal thickness for nasal augmentation and determining the ideal sites for fix-device placement [1, 3]. As rhinoplasty and osteotomies become more common, it is necessary to know the ethnic differences not only in nasal bone shape, but also in the bony structures [1]. Using an osteotome of an inappropriate size can contribute to excessive intranasal soft tissue trauma, resulting in destabilization, excessive hemorrhage, ecchymosis, and postrhinoplasty aesthetic deformity and asymmetry [10]. Proper selection of an osteotome preoperatively (or having the option to change it intraoperatively) increases the success of surgery and preserves the maximum amount of mucosa and periosteum [10]. However there are no studies on the thicknesses of nasal bone on Iranian people. CBCT was introduced to promise low-radiation doses with enough image quality, as well as fast processing and lower cost [11, 12]. The reliability of linear measurements obtained by CBCT was approved in previous researches, concluding that this measurement capability of CBCT machine is reliable for structure closely associated with dentomaxillofacial imaging [13, 14]. The aim of the present study was to have normative data of nasal bone thickness before reconstructive surgery and nasal augmentation, suggesting the guidelines for choosing the appropriate size of osteotome for the selected Iranians through radiography analysis of nasal bone thickness, and to evaluate climate influence and gender on nasal bone shape and thickness. This data can be used as the reference before surgeries.

## 2. Methods and Materials

### 2.1. Patients

In the present descriptive-analytical study, a total of 74 CBCT images of patients who underwent 3D maxillofacial CBCT examination in 2012-2013 were randomly selected from the recorded archive. All CBCT examinations were performed in the relevant conditions (mA 10 to 42 according to patients' size, effective radiation time between 2 to 6 seconds, voxel size 0.15 mm) in Isfahan University of Medical Sciences, Oral and Maxillofacial Radiology Department. Patients with a history of nasal surgery, facial trauma, or known congenital anomaly were excluded from the study. 37 men (with the age range of 19–69, mean age of 37) and 37 females (with the age range of 20–64 and average age of 34) were examined.

### 2.2. Radiographic Measurements: Measuring the Nasal Bone and Pyriform Aperture

**Figure 1:**
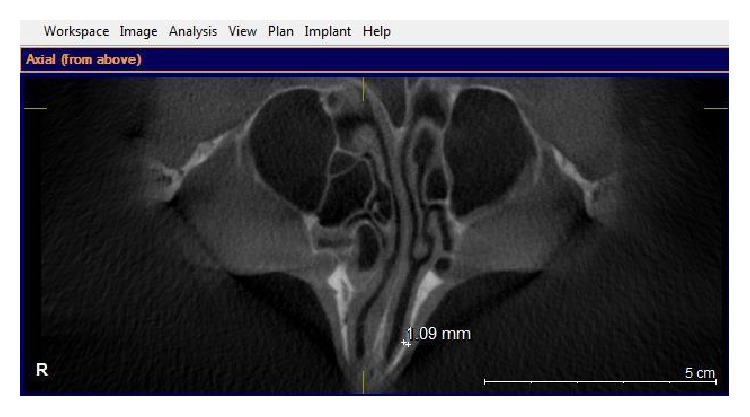
Line mark the lateral osteotomy nasal bone thickness.

**Figure 2:**
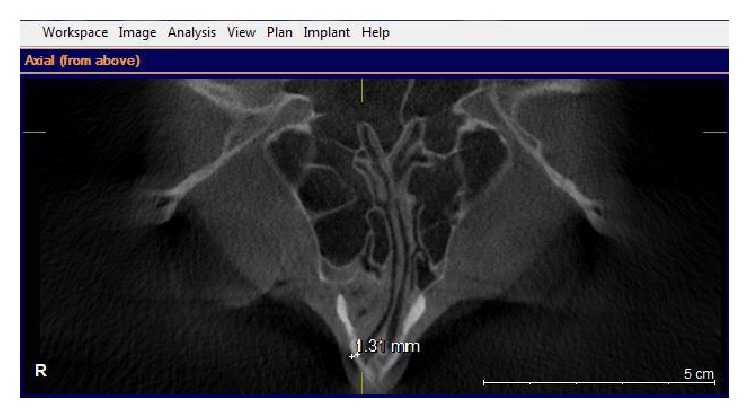
The medial osteotomy nasal bone thickness is shown.

**Figure 3:**
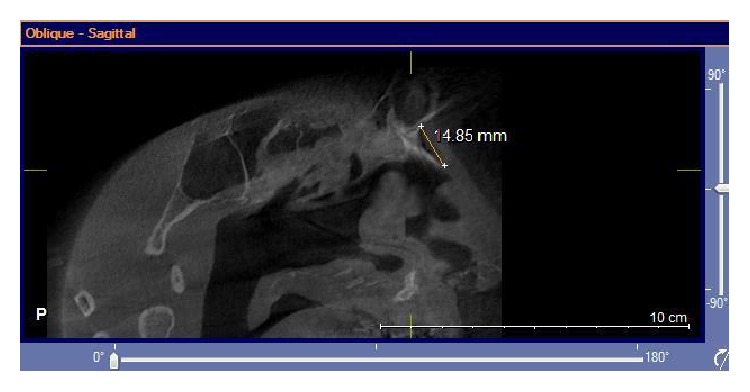
The nasal bone length from the frontonasal suture to the end point of the nasal bone in sagittal view is shown.

**Figure 4:**
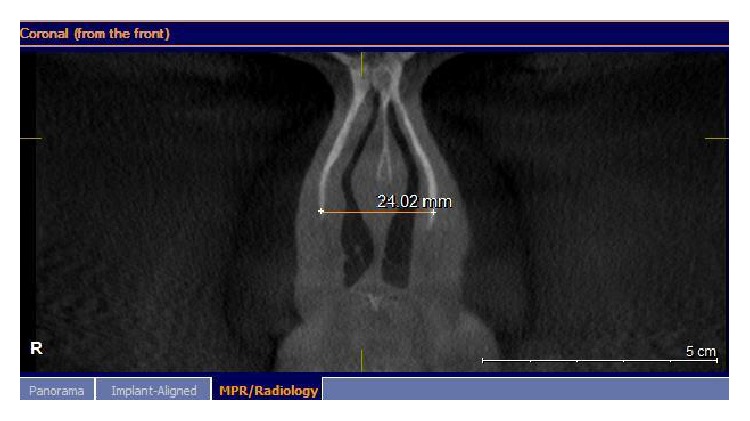
The width of nasal bone aperture was measured at the widest points in coronal view is shown.

Images were taken by using GALILEOUS Comfort 3D imaging system (Sirona Dental System Inc., Bensheim, Germany), to provide standard radiography, and the patients were held in the image field through using occlusal bite block between their teeth (according to the manufacturer's instructions). The system light localizer, which shows the midsagittal line, was also applied. Imaging was performed at 7 mA (42 mAs) and 85 Kvp, with 14-second scan time and 270 rotations. Each scan produced 200 projections in a 15 × 15 × 15 cm field of view, a charge couple device detector, with 1024 × 1024 matrix and 0.15 voxel size. Images were saved in SVG file format and reconstructed using GALAXIS viewer software ver. (GAX5). Then axial, coronal, and sagittal images, as well as 3D model, were reviewed to determine the location of rhinion and nasion. The nasal bone thickness was measured at the sites of the lateral and medial osteotomy lines. The nasal bone thickness was assessed in the axial plane, through the rhinion. First, the nasomaxillary suture was identified. A measuring tool was used to measure the nasal bone thickness at this point. This was termed lateral osteotomy nasal bone thickness (Figure 1). A similar measurement was made at the point, halfway between the rhinion and nasomaxillary suture. This was called medial osteotomy nasal bone thickness (Figure 2). We also measured the nasal bone length from the frontonasal suture to the end point of the nasal bone in the sagittal view (Figure 3). The width of nasal bone aperture was measured at the widest points in coronal view too (Figure 4). The distance measurements were done twice with one-month interval by the first observer and once by the second observer.

### 2.3. Statistical Analysis

The statistical analyses were performed by SPSS software version 20. Intraclass correlation coefficient (ICC) was used to analyze intraobserver and interobserver reliability of measurements (*α* = 0.05). Finally one sample *t*-test was performed for comparing dimensional characteristics of the selected Iranians' nasal bones with the acceptable 0.5 mm mean absolute error (*P* value < 0.05). We expressed the thickness by the mean and standard deviation.

## 3. Results

### 3.1. Measuring the Nasal Bone and Pyriform Aperture

According to ICC value, the interobserver correlation was 0.966 (*P* value < 0.001) between two intervals made by the first observer and the intraobserver correlation was 0.995 (*P* value < 0.001) between the first and the second observer. The mean length of the nasal bone was 23.5 + 3.34 mm in females and 25.7 ± 2.96 mm in males. The mean width of pyriform aperture was 23.77 ± 2.58 mm in females and 25.67 ± 1.79 mm in males (Table 1). Nasal bone length and width of pyriform aperture had no significant differences between the genders (Table 2).

### 3.2. Bone Thickness along the Track of Lateral and Medial Osteotomy

The mean ± SD lateral osteotomy nasal bone thickness was 1.92 ± 0.29 mm in females and 1.73 ± 0.32 mm in males. The medial osteotomy nasal bone thickness was 1.63 ± 0.47 mm in females and 1.94 ± 0.19 mm in males (Table 1). Medial and lateral osteotomy nasal bone thickness had no meaningful differences among the genders.

## 4. Discussion

The most frequent surgical procedures performed by plastic surgeons are nasal bone reconstruction and rhinoplasty, while osteotomy was performed blindly with only physical examinations. Information concerning the nasal bone and pyriform aperture is quite important, and we ensure better performance during surgery by preoperative evaluation of nasal bone anatomy [15, 16]. Few studies have been published on different races. Hwang et al. studied 88 dried skulls from Korean adults and measured the height and width of pyriform aperture [15]. Height of nasal bone was 25.9 + 3.8 mm in males and 24.5 + 3.7 mm in females. The width of pyriform aperture was 25.7 + 1.7 mm in males and 25.4 + 2.1 mm in females [15]. Lang and Baumeister reported that German nasal bone length was 24.9 + 3.2 mm and width of pyriform aperture was 23.6 + 1.8 mm [17]. Ofodie has studied 20 skulls, 6 skulls from Ashant tribe in West Africa, 5 black American skulls, 5 Austrians in northern Europe, and 4 American Indian people. Ofodie reported that the mean nasal bone length of the Ashantis was 21.8 mm, 30.2 mm for Austrians, 30 mm for American Indians, and 27.9 mm for Black Americans. The width of pyriform aperture of the Ashantis was 26.5 mm, 21.6 mm for Austrians, 25.2 mm for American Indians, and 23.4 mm for Black Americans [16]. Ofodie concluded that Austrians' nasal bones were the longest and the Ashantis had the widest pyriform aperture (oval shape) [16]. Karadag et al. studied 80 Anatolian patients and reported the mean nasal bone length of 30.6 mm in males and 29.01 mm in females. The mean width of pyriform aperture was 18.83 mm in males and 18.19 mm in females. It is concluded that the mean length of nasal bone is longer and the width of pyriform aperture is smaller in Anatolian people than in Koreans, Austrians, and Germans [3]. Based on comparing various relevant studies, Anatolian widths of pyriform apertures were the narrowest whilst the Ashantis' were the widest. The width of pyriform apertures of Iranian people was narrower than the Ashantis' and American Indians' and somewhat equal to the Koreans' and Germans' and was wider than the Black Americans', Austrians', and Anatolians'. The Ashantis' nasal bones were the shortest. This data was consistent with the climate influence [16]. The height of Iranians' nasal bones in this study was shown to be shorter than Anatolians', Austrians', American Indians', and Black Americans' and somewhat equal to the Koreans' and Germans' and longer than the Ashantis. Since rhinoplasty is performed more frequently, it is essential to measure the average shape of the nose. This information can be helpful in the clinical field of ENT specialists, for anthropological researches [1]. Osteotomies are performed blindly by using tactile guidance. There is a risk of injuring the supporting mucosa and perichondrium. Obtaining adequate mobilization of bony skeleton is necessary while minimizing the trauma. Excessive damage can lead to postoperative destabilization, aesthetic deformity, and excessive narrowing [18, 19]. It is not necessary for the osteotome blade to cut the entire thickness of nasal bone, because partial thickness fractures produce microfractures [20, 21], so osteotome is to be smaller than the patient nasal bone thickness. Greenstick fracture mobilizes nasal bones to narrow the lateral wall and enable correction of deviated noses. However, thick nasal bones make greenstick fractures difficult, so full thickness fractures are required [22]. Lee and yang studied 75 Korean people and measured nasal bone thicknesses in medial and lateral osteotomy lines. The mean thickness of lateral osteotomy point was 2.06 + 0.36 mm in males and 1.93 + 0.3 in females. The medial point was 1.75 + 0.38 mm in males and 1.78 + 0.35 mm in females. The mere difference in this study as compared to others was only 0.28 mm [1]. Webster et al. performed CT scan on 8 patients. The mean lateral osteotomy nasal bone thickness was 2.39 + 0.68 mm and medial point was 1.18 + 0.3 mm. The difference was 1.21 mm. Hence, more power in lateral point should be considered [19]. Lee et al. studied 100 CT of Asian patients to suggest a guideline for appropriate osteotome selections. The average bony thickness along lateral osteotomy line at middle level was 2.75 + 0.76 mm and was 2.54 + 0.31 mm along the medial osteotomy point. This study showed that trauma to soft tissues in Asian bodies can be minimized by using 2.5 or 3 mm osteotomes [10]. Karadag et al. studied 80 patients, whose mean nasal bone thicknesses were 1.85 + 0.32 mm in lateral and 2.08 + 0.17 mm in medial osteotomy line in males and the values showed 2.04 + 0.17 mm in lateral and 1.091 + 0.46 mm in the medial osteotomy point. The difference was only 0.31 mm [3]. The mean nasal bone thickness in the present study was 1.79 mm and equal power in both points is needed. Thick nasal bones may need the use of a larger osteotome. There were no significant differences in nasal bone thickness, width of pyriform aperture, and length of nasal bone between the genders in the present research and the data was consistent with other studies.

## 5. Conclusions

The present study provided statistical data for nasal bone in the selected Iranian people. These results can provide a guideline for choosing appropriate size of osteotome contributed to the reduction of postoperative complication associated with osteotomy.

## Figures and Tables

**Table 1:** Mean differences between the measurements with the genders. There were no significant differences.

	Male	Female	Absolute difference	*P* value
Medial osteotomy line thickness	1.94 ± 0.19	1.63 ± 0.47	0.31	0.31
Lateral osteotomy line thickness	1.73 ± 0.32	1.92 ± 0.29	0.19	0.39
Nasal bone length	25.7 ± 2.96	23.5 ± 3.34	2.2	0.11
Pyriform aperture width	25.67 ± 1.79	23.77 ± 2.8	1.9	0.25

**Table 2:** Relations between all parameters measured in the present study.

Variable	Width	Height	Medial osteotomy	Lateral osteotomy	Age
Width	1	0.16	0.20	0.030	0.13
Height	0.16	1	−0.09	0.06	0.09
Medial osteotomy	0.20	−0.09	1	0.002	0.11
Lateral osteotomy	0.03	0.06	0.02	1	−0.03
Age	0.14	0.09	0.11	−0.03	1

## Limitations

The primary limitation of the present study was the small sample size and so we did not use a control group. Patients were selected from people having the 3D maxillofacial CBCT examination in Isfahan University. This was the second limitation of the study. Additional multicentric studies are necessary to apply the data about the nasal bones of normal participants among different races. The other limitation of the present study was that the measurements were obtained by using mouse cursor. However, for reducing the errors, the data was measured by two observers.

## Suggestions

The present study suggests preoperative evaluation of nasal cavity by using CBCT (low radiation dose, capability of performing CBCT in small field of view) for reducing postoperative side effects, through using guidelines for choosing suitable size of the relevant osteotome.
